# From Basic Science to Clinical Research to Develop New Solutions to Improve Diagnoses and Treatment of Bladder Cancer Patients

**DOI:** 10.3390/jcm9082373

**Published:** 2020-07-25

**Authors:** Marco Moschini

**Affiliations:** 1 Department of Urology, San Raffaele Scientific Institute, Urological Research Institute, 20132 Milan, Italy; marco.moschini87@gmail.com; 2 Luzerner Kantonsspital, Spitalstrasse, CH-6000 Luzern, Switzerland

Bladder cancer (BCa) is the tenth most common form of cancer worldwide, with 549,000 new cases and 200,000 deaths estimated in 2018 [1]. To address the several unmet questions in the field of BCa research, recently the European Association of Urology (EAU) and the European Society of Medical Oncology (ESMO) selected a panel of experts to define important topics in the field of BCa and to propose possible management solutions [2,3]. In this Special Issue on outcomes and therapeutic management of bladder cancer, we collected a series of articles treating some of the most important topics for the urological community. First, the use of robotic surgery in the treatment of BCa is rapidly increasing, surpassing the use of open surgery in tertiary referral centers [4]. In this regard, literature reporting the efficacy of this technique, in comparison to the old standard, is rapidly increasing [5]. In this issue, we found in a big multicenter collaboration the equivalence of open versus robotic radical cystectomy (RC) in the treatment of BCa patients [6]. Second, functional outcomes after radical cystectomy need to be further reported and investigated to increase the quality of life of BCa patients. Tuderti et al. [7] reported their experience of patients treated with sex sparing robot-assisted radical cystectomy in female patients receiving an intracorporeal neobladder reporting good oncological and functional outcomes 12 months after treatment. From the same institution, Claroni et al. [8] reported on recovery outcomes from anesthesia after robotic-assisted RC.

Third, basic science needs to increase the outcome classification of BCa and the efficacy of diagnostic strategies for an early diagnosis in patients with their first episode of BCa and promptly diagnose a recurrence of BCa in patients who have been already treated. Kim et al. [9] and Sikic et al. [10] and Montero-Reis et al. [11] proposed with different techniques potential markers and therapeutic targets that could improve clinical practices in the future. Fourth, the careful evaluation of variant histology can impact survival outcomes and similarly define optimal treatment strategies by proposing different diagnostic and therapeutic approaches in those patients affected by non-urothelial BCa tumors [12]. Zhou et al. [13] reported on survival outcomes of patients affected by clear cell adenocarcinoma, finding poorer prognosis compared to urothelial cancer. These results confirmed previous findings on this topic [14]. Finally, in this regard the impact of local surgery on patients affected by metastatic BCa is one of the new studied areas in this field [15,16]. I would like to thank the editorial office, authors, reviewers and all the readers for their efforts in putting together this series.
